# A fast and robust hepatocyte quantification algorithm including vein processing

**DOI:** 10.1186/1471-2105-11-124

**Published:** 2010-03-10

**Authors:** Tetyana Ivanovska, Andrea Schenk, André Homeyer, Meihong Deng, Uta Dahmen, Olaf Dirsch, Horst K Hahn, Lars Linsen

**Affiliations:** 1 Jacobs University, Bremen, Germany; 2 Fraunhofer MEVIS, Institute for Medical Image Computing, Bremen, Germany; 3 University Hospital, Essen, Germany; 4 German Heart Institute, Berlin, Germany; 5 Institute of Community Medicine, Ernst-Moritz-Arndt University, Greifswald, Germany

## Abstract

**Background:**

Quantification of different types of cells is often needed for analysis of histological images. In our project, we compute the relative number of proliferating hepatocytes for the evaluation of the regeneration process after partial hepatectomy in normal rat livers.

**Results:**

Our presented automatic approach for hepatocyte (HC) quantification is suitable for the analysis of an entire digitized histological section given in form of a series of images. It is the main part of an automatic hepatocyte quantification tool that allows for the computation of the ratio between the number of proliferating HC-nuclei and the total number of all HC-nuclei for a series of images in one processing run. The processing pipeline allows us to obtain desired and valuable results for a wide range of images with different properties without additional parameter adjustment. Comparing the obtained segmentation results with a manually retrieved segmentation mask which is considered to be the ground truth, we achieve results with sensitivity above 90% and false positive fraction below 15%.

**Conclusions:**

The proposed automatic procedure gives results with high sensitivity and low false positive fraction and can be applied to process entire stained sections.

## Background

Quantification of different cell types in histology is important. For example, quantification of a defined cell type is necessary for determination of the hepatocyte proliferation index to describe the kinetics of a liver regeneration process. Traditionally, observers count cells manually in small regions of interest (ROIs) during microscopical observation. This procedure is very time-consuming and requires an experienced observer, who must be trained to discriminate the target cells from the other cell types. In our case, we are interested in discriminating hepatocytes, the functional parenchymal cells in the liver, from non-parenchymal cells of the liver.

Recently, with the availability of digital photography the computer-assisted cell counting has gained popularity. The observer marks each cell to be included using image analysis tools, e. g., GIMP http://www.gimp.org/ or Image Tool http://ddsdx.uthscsa.edu/dig/itdesc.html, and the marked events are enumerated. The image overlaid with marked target cells is saved for documentation. There exist also semi-automatic and automatic solutions based on image analysis systems used in clinical routine. For example, our project group recently presented a macro based on a commercially available software http://industrial-microscope.olympus-global.com/en/ga/product/analysisfive/[1].

Such solutions based on the analysis of small 2D samples from a large 3D object suffer from the sampling bias problem. The analysis of small 2D samples is only valid, if target events are distributed homogeneously in the whole 3D object. This assumption does not hold generally for liver regeneration, as this process is subject to local regulation. Spatial distribution of proliferating hepatocytes within the smallest functional liver unit, the lobules, depends on the hepatic zone and can vary substantially throughout the liver. Hence, the entire 3D object needs to be looked at, which is, again, a tedious and time-consuming effort when keeping the user in the loop.

**Figure 1:**
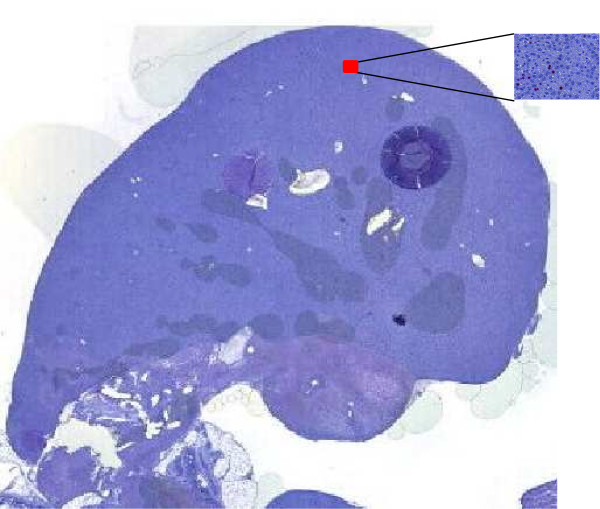
**Whole stained section**. Whole stained section digitized with a resolution of 53248 × 52736. The red rectangle shows a selected ROI with resolution of 2576 × 1932.

The ultimate solution to this problem and our overall project goal is to subject serial sections of the whole sample to an automatic quantitative assessment. The first step towards this full automatization is to detect the proliferation index, i. e., the ratio of the number of proliferating cells and the overall number of cells, in whole sections of the rat liver; an example image is shown in Figure 1. To accomplish this goal a series of tasks needs to be tackled. First, the zones of interest containing hepatocyte information must be defined. Second, due to the sample size it has to be divided into smaller parts. Third, the parts containing hepatocyte information has to be processed, i. e., the nuclei must be detected in each image. Fourth, the nuclei quantification information must be accumulated for the whole section.

In this paper, we address the hepatocyte quantification task. The specific aim of this task was to develop an automatic approach, that is fast, robust to different image appearances, and allows to analyze batches of images without additional user interaction.

In recent years, a number of sophisticated automatic image processing approaches for histological sections have been proposed [2]. However, it is difficult to compare them to each other due to the difference of staining methods applied to the data and the related image analysis problems. There exist several popular directions in segmentation of microscopic structures. They include fuzzy clustering [3], parametric and geometric deformable models [4,5], morphological watershed-based approaches [6,7]. Though the variety of the proposed methods is huge, most of them are aimed to detect the boundaries of the nuclei cells as precisely as possible, which is actually not needed for our purposes. The complexity and computational costs of these methods are not necessary and not justified in our case. In addition, the methods have either problems with overlapping nuclei or are strongly dependent on the data staining. Our task was to develop an approach that is fast, robust to different data appearances within the staining specific to our project, and whose aim is not to detect cell boundaries but rather to evaluate the number of cells, in particular, that it can deal with overlapping cells appropriately.

We recently presented a preliminary automatic approach for quantifying hepatocytes in normally regenerating rat livers [8]. The proposed processing pipeline consists of four main steps. First, the data is smoothed. We tested and compared different smoothing filters to find the most appropriate for the given task. Here, we make use of this investigation by incorporating the most suitable one in the pipeline presented in this paper. Second, we applied an automatic thresholding method similar to the one described by Petushi et al. [9]. The applied method was suitable for the data we had used. However, to build a method that is robust against unavoidable variations in staining intensities and that can handle the occurrence of vein structures, we had to develop a new thresholding strategy, which is presented in this paper. Third, a detection step of structures of certain size and shape is applied. And finally, we applied a Hough transformation step, which showed to be effective when dealing with overlapping nuclei and computationally reasonable when the search space is reduced. This final step is similar to the compact Hough transformation-based approach [10].

The main limitation of our algorithm [8] was that it was not able to handle the presence of the vein structures in the images. In this paper, we now propose an improved automatic pipeline that includes an appropriate vein structure handling.

## Methods

Liver samples of about 0.5-1 *cm*^3 ^in size from rats subjected to 70% liver resection were formalinfixed, paraffin-embedded, and used for cutting histological sections of 4-6 *μm *thickness. Thereafter, a special immunohistochemical procedure [1,11], namely BrdU-staining, was applied to them. As a result, nuclei of dividing (proliferating) cells, hepatocytes, and other non-parenchymal stromal cells are marked in red, whereas the nuclei of the non-dividing cells are marked in blue.

Digitized images of the stained sections are taken at a 200-fold magnification. An example of such an image is shown in Figure 1. The rat liver consists of parenchchymal cells (hepatocytes) and non-parenchymal cells (for instance, bile duct cells, Kupffer cells, sinusoidal endothelial cells, lymphocytes). According to the portal blood flow, the hepatic parenchyma is divided into 3 zones. Zone 1, also called portal zone, is surrounding the portal tract (PT), a complex histological structure consisting of several vascular components such as a portal vein, a hepatic artery and several bile ducts embedded in histiocytic cells and connective tissue. Zone 2 is surrounding the central vein (CV), which is draining the smallest functional unit of the liver, the hepatic lobule. Zone 3 is the midzonal area between zone 1 and zone 2. Despite their anatomical and functional differences, for excluding areas with non-parenchymal structures from further proliferation analysis portal tract and central vein are considered to be similar structures and are referred to as "venous (vessel) structures" throughout the text. ROIs with a resolution of 2576 × 1932 pixels are selected from these three liver zones.

We have made a series of tests on images from eight different datasets. Each dataset represents ROIs from the liver samples of one animal. These datasets belong to two groups that had different contact time of the section with staining solutions, which resulted in variations in image contrast. The datasets within each group are also subject to variations in color properties, which occur due to some differences in the histological processing of the tissue sample and may also occur during image acquisition (camera settings).

We keep the following naming convention: each image name has a number that denotes the dataset. For example, D1 denotes the image from liver zone 3 of dataset 1. PT and CV in the names belong to the images that were taken from the liver zones 1 and 2. Liver zones 1 and 2 contain vessel structures.

## Results

### Algorithm

To solve the task of automatic processing of the ROIs of histological sections, we have developed an algorithm and created a tool which can assess the total number of events (total hepatocyte nuclei) as well as the number of positive events (proliferating red-stained hepatocyte nuclei). It calculates the relative proportion of the positive events, namely the ratio between proliferating and total hepatocytes (BrdU-LI) automatically in one "processing run". To do so, it eliminates morphological structures impairing the analysis (central vein or portal tracts). It creates a batch calculation allowing to analyze several images without user interaction, which forms the basis for evaluating a whole section. Moreover, it facilitate the validation by creating a tool for determining statistical measures of quantification quality, namely sensitivity and false positive fraction.

**Figure 2:**
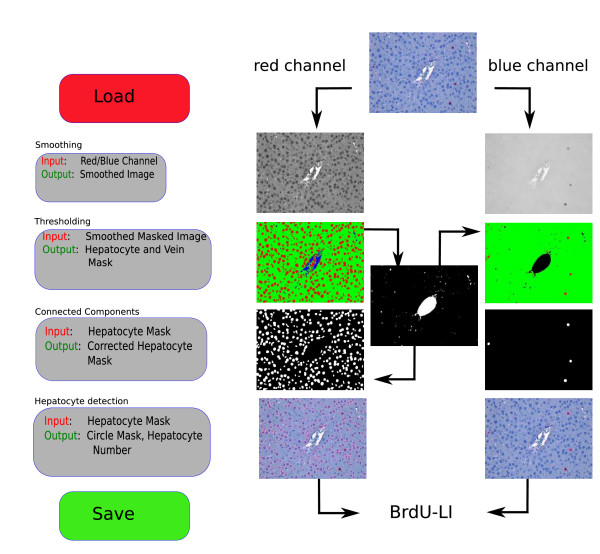
**Pipeline**. Pipeline for automatic hepatocyte quantification. Our processing pipeline consists of four main steps. The flowchart is depicted on the left-hand side; the impact of the individual steps on the right-hand side. The detection of total HC-nuclei in the red image channel and proliferating HC-nuclei in the blue image channel is shown side by side. The vein region is smoothed and eliminated. For proliferating HC-nuclei detection, the vein mask is excluded in the thresholding step.

The processing pipeline extends the one described in our previous work [8]. The main contributions of the extended pipeline include the processing of images containing vein structures, an improved thresholding step, and an extended connected-components analysis for the detection of the number of total and proliferating hepatocytes. In Figure 2, the flowchart for automatic detection of the proliferation index is depicted on the left-hand side. The result for each processing step is shown next to the correspondent flowchart box on the right-hand side.

#### Smoothing

Based on the applied staining technique, it can be observed that all nuclei are visible in the red color channel, whereas the proliferating cells are distinguishable in the blue color channel. We apply a smoothing step to the representative image channels to reduce noise. As previously reported [8], we selected the bilateral filter as one of the most suitable algorithms for the current task. There are different implementations of the bilateral filter in the literature [12-14]. We investigated two variants, which are compared in the Discussion section.

#### Automatic Thresholding

We previously used Otsu thresholding [15] to automatically separate nuclei from the background in the smoothed image [8]. The original Otsu method performs histogram-based image thresholding. It assumes that the image contains two classes of pixels (foreground and background) and calculates the optimal threshold that separates the classes, such that their within-class variance is minimized.

The original binary thresholding is not sufficient for processing images containing vascular structures [8], as there are not only two, but three classes in such images, namely nucleus, vein, and background. For such a purpose, the multi-level Otsu thresholding can be used [16].

Our experiments showed that applying multilevel Otsu thresholding to our segmentation task has some severe drawbacks (see Discussion section). Thus, we decided to replace it by an automatic thresholding step based on expectation maximization (EM) [17]. Let ***y ***denote incomplete data consisting of observable variables, and let ***z ***denote the missing data. ***z ***can be either missing measurements or a hidden variable that would make the problem easier if its values were known. ***y ***and ***z ***together form the complete data. Let *p*(***y***, ***z***|*θ*) denote the joint probability density function of the complete data with parameters given by vector *θ*. The EM consists basically of two steps: expectation and maximization.

In our case, we consider the Gaussian mixture model for the EM algorithm. It assumes that the image pixel intensities *y*_1_, ..., *y*_*m *_∈ ℝ are samples of independent observations from *n *Gaussian distributions with unknown parameters. Let *z*_*j*_∈ {1, 2, ..., *n*} denote the index number of the Gaussian distribution that *y*_*j *_has been drawn from.

The probability density function of the *i*-th one-dimensional Gaussian distribution is(1)

where *θ *= {*μ*_1_, ..., *μ*_*n*_, *σ*_1_,..., *σ*_*n*_, *p*(***z ***= 1), ..., *p*(***z ***= *n*)} with *μ*_*i *_and *σ*_*i *_being the mean and the variance of the *i*th Gaussian distribution, and *p*(*z *= *i*) being the *i*th class membership probability (or proportion). The class proportion is the probability for each Gaussian distribution being drawn from all observations.

The log-likelihood of the joint event that is to be maximized can be written as(2)

Before the first iteration, one must find initial estimates of *μ*, *σ*, and the class proportions. To compute the initial *μ*, the histogram is divided into *n *equal parts and *μ*_*i *_is taken as a mean of each part. The initial *σ*_*i *_are taken to be equal to the maximum value in the image. The initial class proportions are equal to . Then, the main goal is to identify the unknown distribution parameters *θ*.

In the expectation step, the conditional distribution with respect to the current unknown parameter estimates is computed by(3)

where *θ*_*t *_is the estimation of the parameters on iteration *t*, *p*(*y*_*j*_|*z*_*j *_= *i, θ*_*t*_) = *N *(*μ*_*i*_, *σ*_*i*_) is the Gaussian probability density function at *y*_*j*_, and *p*(*z*_*j *_= *i*| *θ*_*t*_) is the probability of the class *i *for *y*_*j*_. The values of *μ*, *σ*, and *p *are taken from the previous maximization step.

In the maximization step, the values of *μ*, *σ*, and *p *which maximize the log-likelihood are re-estimated:(4)

To speed up the computations, we assume not the image pixels, but the image histogram values to be *y*. Thus, the corresponding probabilities for *y*_*j *_are taken into account in each expectation step when computing *p*(*y*_*j*_|*z*_*j *_= *i, θ*_*t*_).

When the difference between the log-likelihood values found in two iterations is lower than a certain accuracy value, the algorithm stops. The algorithm execution time varies depending on the selected accuracy. For our application, an accuracy value of 10^-4 ^was enough to obtain the desired results in a short amount of time.

In the current implementation, the choice of the number of classes in the image is indicated to our tool using a filename convention. The filenames must indicate, which files contain vein structures. After the automatic thresholding is completed, we obtain the proper mask that consists either of two or three classes. We place each class in a separate image channel and combine the RGB mask image (see Figure 2).

#### Connected Components Processing

To discriminate the candidates for hepatocytes from all other structures of the mask image, we apply some standard algorithms, namely morphological operations [18] and size and roundness filters.

First, the morphological operation of erosion [18] shrinks the regions to separate "weakly connected" regions, i. e., regions with a thin and fragile connectedness. Second, the Fill Holes filter [18] is applied to erase potentially existing holes in regions and, hence, make the regions simply-connected. Third, we use connected-component labeling in the image [6] to analyze each connected component according to its area, i. e., we threshold the connected components according to their area and the respective equivalent diameter. The equivalent diameter *d*_*eq *_is the diameter of a circle with the same area as the connected component, i. e.,(7)

where *A *denotes the area of the connected component. Then, the dilation operation [18] can be applied to expand the regions and restore the original region sizes. Finally, we eliminate the non-round regions by excluding the connected components that have a form factor lower than a certain threshold. The form factor *F *given by(8)

is equal to 1 for circles and approaches 0 for elongated regions, where *A *is again the area of the connected component and *P *is its perimeter. Perimeter *P *is computed by summing the distances of all neighboring pairs of pixels along the border of the connected component.

In case there are vein structures present in the image, those are eliminated using the vein structure mask obtained by the thresholding step. We compute the ratio between the area of the largest vein component and the whole area covered by vein, then smooth the vein region according to that ratio, and subtract the resulting mask from the hepatocyte channel mask. Subtracting the mask means that we set all those pixels to black in the hepatocyte mask that are white in the vein mask, see Figure 2. The smoothing is needed to connect disjoint vein regions and to exclude inner part of the vein. To smooth the vein mask we, again, apply a bilateral filter.

Such a vein exclusion technique can handle vein structures that consist of several disconnected regions lying close together. If the vein region consists of one major connected component, smoothing is not necessary and can be omitted. However, such a connected vein regions may contain some blood cells, which appear as holes in the vein structure mask. A Fill Holes filter [18] takes care of removing the blood. In the current implementation, the type of the vein exclusion is defined by a parameter in the pipeline settings.

The vein exclusion is applied to the red image channel during the detection of the whole number of HC. In the blue image channel the vein mask found in the red channel is discarded in the thresholding step, see Figure 2.

#### Hepatocyte Detection

To calculate the number of circular, possibly overlapping regions in the image, we utilize the Hough transformation [19], looking for circles within a pre-defined radius interval. The algorithm operates in the parameter space of the circles. The search for circles stops when the height of the currently detected maximum in the accumulator matrix is smaller than a certain percentage of the first detected maximum.

In a post-processing step, we analyze the obtained circle list. We exclude all those circles whose center lies outside the image or does not lie inside the hepatocyte region mask. Moreover, from a group of circles lying close together we exclude the ones having less overlap with the hepatocyte region mask. The closeness of the circles is defined as(9)

where *c*1, *c*2 denote the centers of the circles, *r*_1_, *r*_2 _denote the circles' radii, and *F*_*cl *_is a user-defined parameter.

All the above-mentioned algorithms have been implemented, using MeVisLab, Software for Medical Image Processing and Visualization (see http://www.mevislab.de).

### Testing

#### Inter-observer performance

As the groundtruth which is considered to be the golden standard is manually defined by experts, we have carried out the inter-observer agreement analysis. We asked four experts to mark total and proliferating HC on one batch of data (D5-8). Moreover, the experts were also supposed to mark all events that could be "non-hepatocytes" and "non-proliferating-hepatocytes". The results are presented in Table 1. Relative standard deviation (which is computed as ) has been calculated for each image and then its mean has been found. The results have shown that the relative standard deviation is 15% for the total number of hepatocytes. Its value is higher for the number of proliferating hepatocytes (20%), which is due to the fact, that, for instance, dataset D5 has very low proliferation rates, hence, the results are very sensitive to expert decisions. We have compared our results to the sets of expert groundtruths and the resulting mean sensitivity is close to 90%. In Tables 2, 3, we show the results obtained with comparison to one of the expert groundtruths.

**Table 1:** Inter-observer variability results.

Inter-observer variability				
	**Total HC**	**Total Non-HC**	**Proliferating HC**	**Proliferating Non-HC**

D5	219	162	4	399
	198	150	4	357
	189	257	2	188

D5-PT1	266	164	2	473
	213	170	3	442
	314	150	4	453
	225	272	2	188

D5-CV1	265	167	2	444
	227	137	2	415
	303	127	1	184
	202	214	1	371

D6-PT1	215	174	90	319
	207	165	103	295
	276	135	107	292
	197	178	77	99

D6-CV1	232	163	81	348
	213	127	78	276
	291	139	92	345
	200	208	62	127

D7	243	134	39	347
	230	153	42	280
	264	103	49	326
	202	191	39	168

D7-PT1	249	146	33	383
	214	113	34	317
	282	116	37	359
	193	251	24	151

D7-CV1	264	153	28	421
	195	124	27	355
	289	122	33	398
	212	214	21	188

D8	237	189	147	284
	216	117	143	200
	254	142	158	242
	203	181	122	242

D8-PT1	242	255	102	402
	200	195	109	321
	274	197	134	348
	187	319	75	388

D8-CV1	230	116	61	315
	185	69	53	235
	253	78	65	273
	190	207	51	398

Mean Relative StdDev	15.02	27.96	20.92	29.42

**Table 2:** Total hepatocyte quantification results for eight different data sets.

Total hepatocytes							
**Image**	**Detected**	**TP**	**FP**	**FN**	**Sensitivity**	**FPF**	**User P**

D1	230	221	9	22	0.91	0.039	243

D1-PT1	210	188	22	20	0.90	0.1	208

D1-CV1	302	269	33	38	0.88	0.1	307

D2	223	201	22	14	0.93	0.09	215

D2-PT1	212	166	46	9	0.95	0.21	175

D2-CV1	230	200	30	17	0.92	0.13	217

D3	299	230	69	10	0.96	0.23	240

D3-PT1	268	206	62	13	0.94	0.23	219

D3-CV1	269	216	53	13	0.94	0.19	229

D4	229	212	17	33	0.87	0.07	245

D4-PT1	216	198	18	15	0.93	0.08	213

D4-CV1	237	218	19	10	0.95	0.08	228

D5	226	189	49	12	0.94	0.21	201

D5-PT1	217	210	7	15	0.93	0.03	225

D5-CV1	237	197	40	5	0.97	0.16	202

D6-PT1	216	186	30	11	0.94	0.13	197

D6-CV1	224	186	38	14	0.93	0.16	200

D7	230	191	39	11	0.94	0.17	202

D7-PT1	236	190	46	3	0.98	0.19	193

D7-CV1	218	204	14	8	0.96	0.06	212

D8	229	197	32	6	0.97	0.13	203

D8-PT1	214	177	37	10	0.95	0.17	187

D8-CV1	208	174	34	16	0.91	0.16	190

Mean					0.93	0.135	

**Table 3:** Proliferating hepatocyte quantification results for eight different data sets.

Proliferating hepatocytes							
**Image**	**Detected**	**TP**	**FP**	**FN**	**Sensitivity**	**FPF**	**User P**

D1	3	3	0	0	1.00	0.00	3

D1-PT1	3	3	0	1	0.75	0.00	4

D1-CV2	4	4	0	0	1.00	0.00	4

D2	22	18	4	1	0.94	0.18	19

D2-PT1	27	23	4	1	0.96	0.14	24

D2-CV1	11	8	3	0	1.00	0.27	8

D3	27	24	3	1	0.96	0.11	25

D3-PT1	12	11	1	7	0.61	0.08	18

D3-CV1	41	39	2	1	0.97	0.04	40

D4	98	96	2	27	0.78	0.02	123

D4-PT1	105	102	3	14	0.87	0.02	116

D4-CV1	58	57	1	7	0.89	0.01	64

D5	3	2	1	0	1.00	0.33	2

D5-PT1	1	1	0	1	0.5	0.00	2

D5-CV1	1	1	0	0	1.00	0.00	1

D6-PT1	90	77	13	3	0.96	0.14	80

D6-CV1	65	61	4	5	0.92	0.06	66

D7	40	37	3	2	0.94	0.075	39

D7-PT1	26	25	1	0	1.00	0.03	25

D7-CV1	24	20	4	1	0.95	0.16	21

D8	138	120	18	2	0.98	0.13	122

D8-PT1	93	72	21	3	0.96	0.22	75

D8-CV1	55	50	5	1	0.98	0.09	51

Mean					0.91	0.09	

However, when the description of the target is rather vague (to mark "non-hepatocytes"), the experts result in much less agreement (around 30%). Therefore, we assume that computation of True Negatives (TN) and, correspondingly, the specificity or the false positive rate brings less value due to such a high inter-observer dispersion. Instead, we propose to evaluate the false positive fraction (FPF) within the total number of detected events, i. e., . Intuitively clear, that results where the false positive fraction is approaching to 0 are preferable, and when the FPF is close to 1, the solution is too inexact.

#### Parameter selection

**Figure 3:**
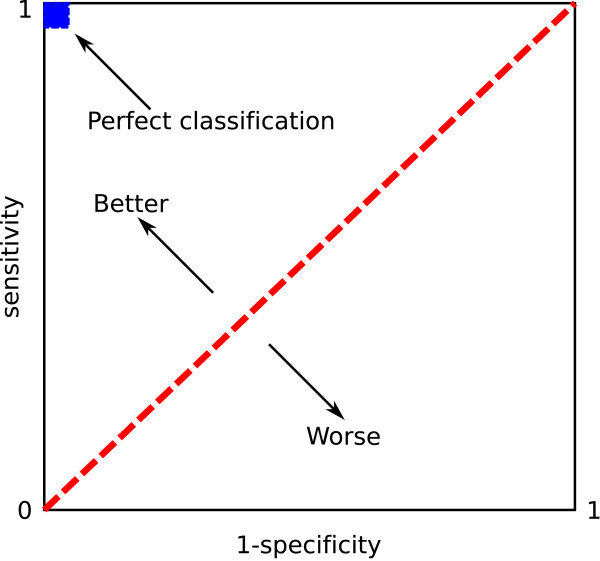
**ROC Space**. A ROC space is defined by 1 - specificity and sensitivity as x and y axes respectively, which depicts relative trade-offs between true positive (benefits) and false positive (costs).

For selection of the parameters in the most optimal way, such tool as receiver operator characteristic (ROC) [20] is very useful. A ROC space is defined by 1-*specificity *and *sensitivity *as *x *and *y *axes respectively, which depicts relative trade-offs between true positive (benefits) and false positive (costs). In Figure 3, the ROC space is shown. The best possible classification method would yield a point in the upper left corner or coordinate (0, 1) of the ROC space, representing 100% sensitivity (no false negatives) and 100% specificity (no false positives). However, as it is shown above, the number of True negatives (TN) is too rough and does not bring meaningful information, hence, we propose to build a ROC-like curve with FPF (false positive fraction) on abscissa. Its behavior will be the same as of the standard ROC-curve.

**Figure 4:**
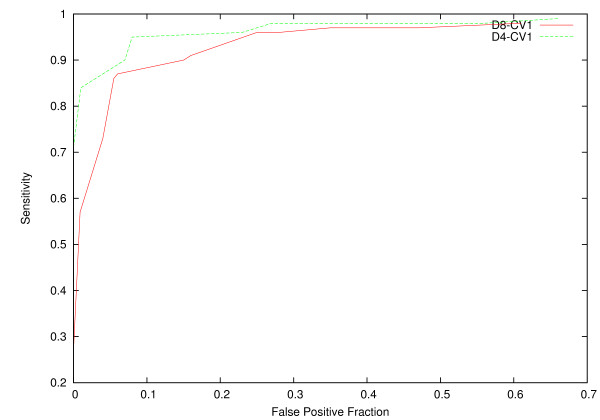
**ROC-like Curve for Hough Transformation Threshold**. ROC-like curve has sensitivity as *x *axis and false positive fraction (FPF) as *y *axis. ROC-like curves are built for images *D*4-*CV *1 and *D*8-*CV *1 for threshold values [0.1, ..., 0.9] with *step *= 0.1. The optimal values of the relevance threshold for Hough transformation lie within interval [0.5, 0.6], which corresponds sensitivity ≥ 90% and FPF ≤ 10%.

One of the parameters that influences strongly the results is the relevance threshold for Hough transformation. In Figure 4, we show the ROC-like curves for detection of the total HC for images D4-CV1 and D8-CV1. The threshold values are [0.1, ..., 0.9] with *Step *= 0.1. We have observed, that the best results (sensitivity is ≥ 90%, FPF is ≤ 10%) are achieved with the relevance threshold interval [0.5, 0.6].

The parameter selection is a trial-and-error process. In general, one should take into account the following data characteristics for the optimal parameter determination: the noise level of images; the size and roundness of the target objects (hepatocytes, in our case); if the target objects can be clumped together, then the size and roundness of the clumped objects; the level of overlap of the single target objects.

Since our smoothing step is followed by an automatic thresholding step, the smoothing must be sufficient, otherwise the thresholding will most likely fail. Otherwise, when the denoising parameters are too high, the cells lying close will be merged together and detected as one connected component, that could be either rejected on the connected component analysis step as a too big or too non-round object (this would cause a number of false negatives) or Hough transformation would detect there a number of false positives. Moreover, to detect the proliferating hepatocytes, i. e., to process the blue image channel, we select a much stronger smoothing. This allows us to leave out non-proliferating hepatocytes, which are much brighter than the proliferating ones, and to separate the image into two classes (hepatocytes and background) in the thresholding step.

While processing the resulting connected components, we empirically measured the size and the roundness of the single HC and the HC that are clumped together. For Hough transformation, we selected the parameters according to the size (HC radii interval), form (relevance threshold and *σ *for the accumulator array smoothing), and the overlap level (closeness factor) of the single HC. If these parameter values are underestimated, then practically all detected components pass, which will cause a significant number of false positives. Otherwise, the overestimated parameter values will cause many misses in the detection.

Overall, for detection of the total and proliferating number of hepatocytes the following parameters are used: for smoothing *σ*_*s *_= 16, *σ*_*r *_= 0.15 and *σ*_*s *_= 50, *σ*_*r *_= 0.1, respectively; for connected component processing *d*_*eq*_∈ [35, 200], *F *= 0.2, and *A *∈ [700, 8000]; and for Hough transformation *r *∈ [14, 50], *σ *= 10, *relevance *= 0.5, and *F*_*cl *_= 0.5 and *F*_*cl *_= 0.75, respectively.

#### Pipeline results

We tested our processing pipeline on the detection of the number of total HC and the number of proliferating HC. The proliferation index detection takes on an Intel(R) Core(TM)2 CPU T7200 @ 2.00 GHz computer with 2 GB DDR2 for one ROI image with a resolution of 2576 × 1932 on average 135 seconds.

The proposed pipeline has been validated using two sets of samples (D1-4, D5-8), which have variations in staining intensity and contrast due to some differences in the histological processing. The ground truth is determined by manual identification of targeted red and blue labeled hepatocytes. We have taken the groundtruth from one of the observers. The result is compared with the cells detected by the application. In order to allow for a fast comparison of the results, a small "validation tool" has been developed, which allows for creating sets of groundtruth and obtaining sensitivity and false positive fraction values.

Our evaluations are presented in Tables 2 and 3. The following notations for the headings are used: "Detected" means the number of circles found by Hough transformation; "TP" is the number of True Positive hepatocytes, which is the result of the overlay of detected circles and the user expectations; "FN" denotes the number of False Negative hepatocytes, which is the difference between the Ground Truth Positives and the True Positives; "FP" stands for the number of False Positive hepatocytes, which is the difference between "Detected" and "TP"; and User P is the number of Positive hepatocytes manually specified by the expert.

The most important numbers are the computed sensitivities and false positive fractions (FPF). Sensitivity is defined by *T P/*(*T P *+ *F N*) and measures the proportion of actual positives, while FPF is defined by *F P/*(*Detected*) and measures the proportion of false positives in the number of detected events.

**Figure 5:**
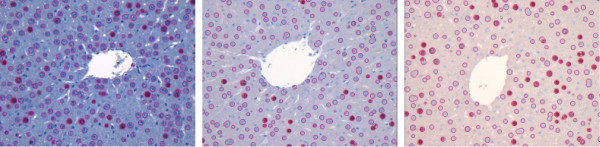
**Example Results**. Result for detection of all hepatocytes for the images shown in Figure 12. No parameter adjustment was done. The resulting statistical measures in form of sensitivity/FPF are D3-CV1: 0.94/0.19, D7-CV1: 0.93/0.06, and D8-CV1: 0.9/0.16. Our pipeline is robust enough to allow accurate analysis of images with different color properties without the need for individual adjustments.

The processed datasets belong to two batches, namely D1-D4 belong to the first batch and D5-D8 belong to the second batch. The second batch of datasets corresponds to the data used in Tables 2 and 3 in previous work [1]. In Figure 5, examples of our results for images from both batches are depicted in an overlaid manner. The red circles are the output of our pipeline. The images visually document the findings in Table 2.

## Discussion

### Smoothing analysis

**Figure 6:**
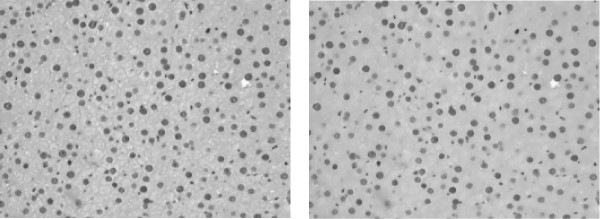
**Smoothing Analysis**. Comparison of the smoothing results obtained with different implementations of the bilateral filter for image D8: Bilateral filter chain (left), fast bilateral filter (right). The bilateral filter chain approach produces much smoother and "round" result, while the fast bilateral filter introduces some distortions due to its implementation with downsampling. The bilateral filter chain detects 200 HC out of user-marked 205, while the fast bilateral filter due to the introduced distortions detects only 192 HC out of 205. However, it does not significantly worsen the detection rates: The bilateral filter chain produces results with sensitivity of 97% and false positive fraction of 13%, while the fast bilateral filter produces results with sensitivity of 94% and false positive fraction of 10%.

We propose to use two different variants for the bilateral filter. One is the bilateral filter chain as implemented by Aurich et al. [12]. It allows for the removal of fine details while preserving the edges of the coarser structures. However, the computational costs of this implementation are rather high. Therefore, we also applied a faster implementation, namely the approximation of the bilateral filter introduced by Paris et al. [14]. The fast approximation is based on downsampling the image, applying the convolution, and upsampling again. Such a technique is favorable in terms of speed, but introduces some distortions in the image for the slight smoothing, which can affect the subsequent automatic thresholding step negatively and may hinder the appropriate nucleus detection. However, we observed that the distortion effect is mostly "corrected" by the connected components post-processing and the detection results for the two implementations are similar. In Figure 6, smoothing results for both bilateral filter implementations are presented.

### Thresholding analysis

**Figure 7:**
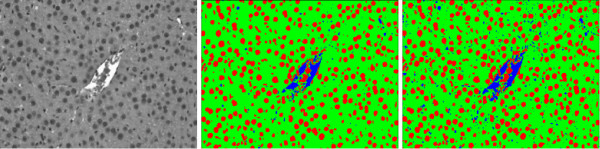
**Thresholding with representative classes**. Otsu thresholding (middle) and EM thresholding (right) applied to the red image channel (left) give similar results if the classes are representative.

**Figure 8:**
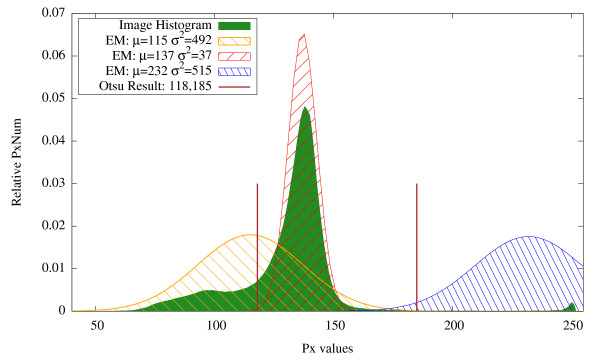
**Thresholding with representative classes: Histogram**. Results of Otsu and EM method overlaid with image histogram for Figure 4.

In general, Otsu thresholding and expectation maximization produce quite similar results with threshold values lying close to each other. For instance, in Figure 7, the initial image and results of both methods are shown. In Figure 8, the threshold values obtained with Otsu thresholding and EM are presented being overlaid with the respective histogram. Otsu thresholding delivers two threshold values, namely 118 and 185, which separates the data range into three regions. The EM method delivers three Gaussian curves, which represent the likelihood of belonging to the three classes, i. e., the class with the highest value is the most likely one. The classification changes where the curves intersect, i. e., intersection points deliver the threshold locations. They are close to the Otsu thresholds (marked with bars).

**Figure 9:**
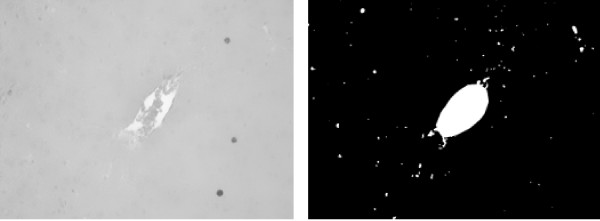
**Non-representative cell class**. Close-up view for the smoothed blue image channel (left) and vein mask (right) for Figure 2. The cell class is not representative in the image.

**Figure 10:**
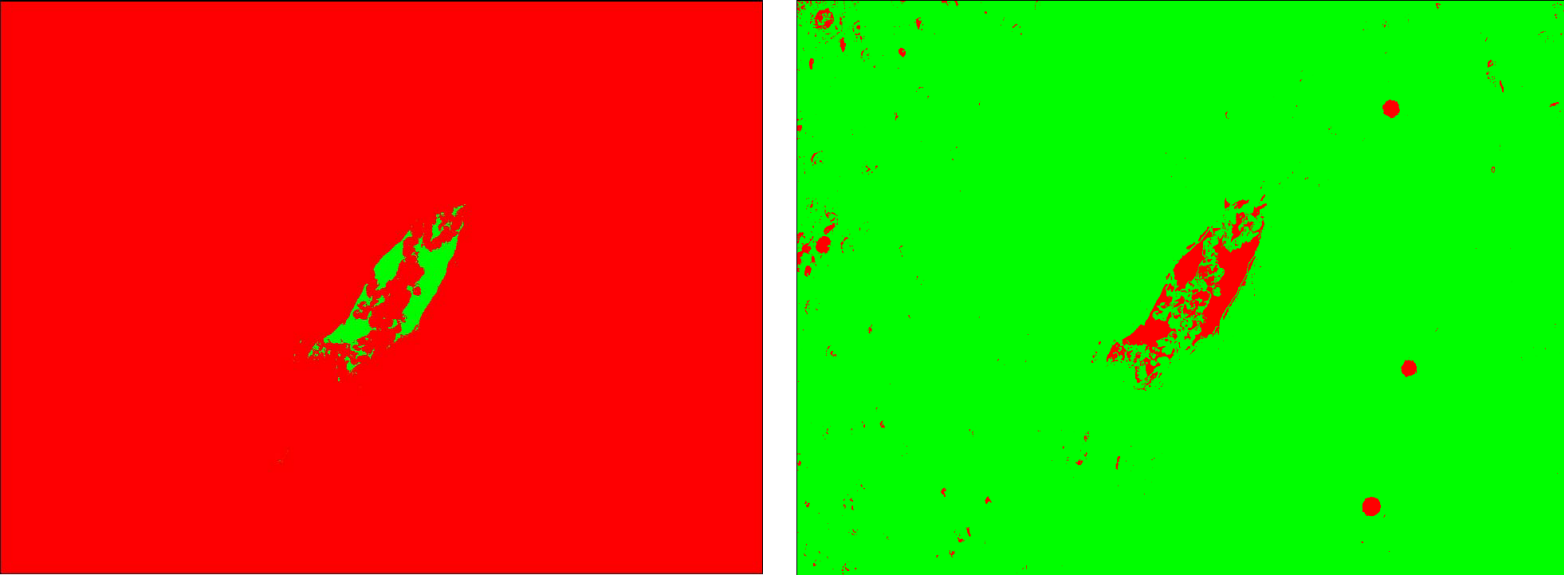
**Thresholding result with vein**. Results for Otsu thresholding (left) and EM (right) methods for the image in Figure 9 without vein exclusion. Otsu thresholding misclassifies the cells, which cannot be restored. EM assigns both cells (darkest spots) and vein (brightest spots) to the same Gaussian distribution. After connected component processing, it is possible to extract the cells from the EM result. However, a number of false positives (in the vein area) is also detected.

However, when one of the classes in the images is not representative, i. e., it is negligible when compared to the other classes, the cell class may be misclassified. For example, for an image with low proliferation rate as the one depicted in Figure 9, the results for Otsu thresholding and EM are shown in Figure 10. Otsu thresholding separates the vein from the background, but the information about the cell class is lost. EM assigns the vein and the cells to the same class, which can lead to some additional false positives detection.

**Figure 11:**
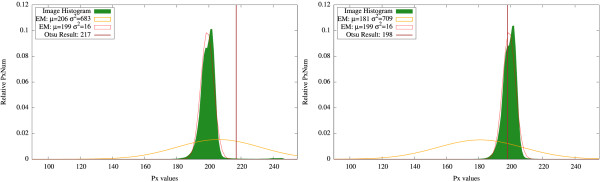
**Thresholding result: Histogram**. Results for Otsu thresholding and EM methods overlaid with image histogram for Figure 9 before (left) and after (right) vein exclusion. When the vein is not excluded (left), Otsu thresholding misses the cell class. With vein exclusion (right), Otsu thresholding is still not able to separate the cell class, as the variations inside the background class are more noticeable. EM assigns in both cases (left and right) all non-representative classes (cells and vein) to the same Gaussian curve.

**Figure 12:**
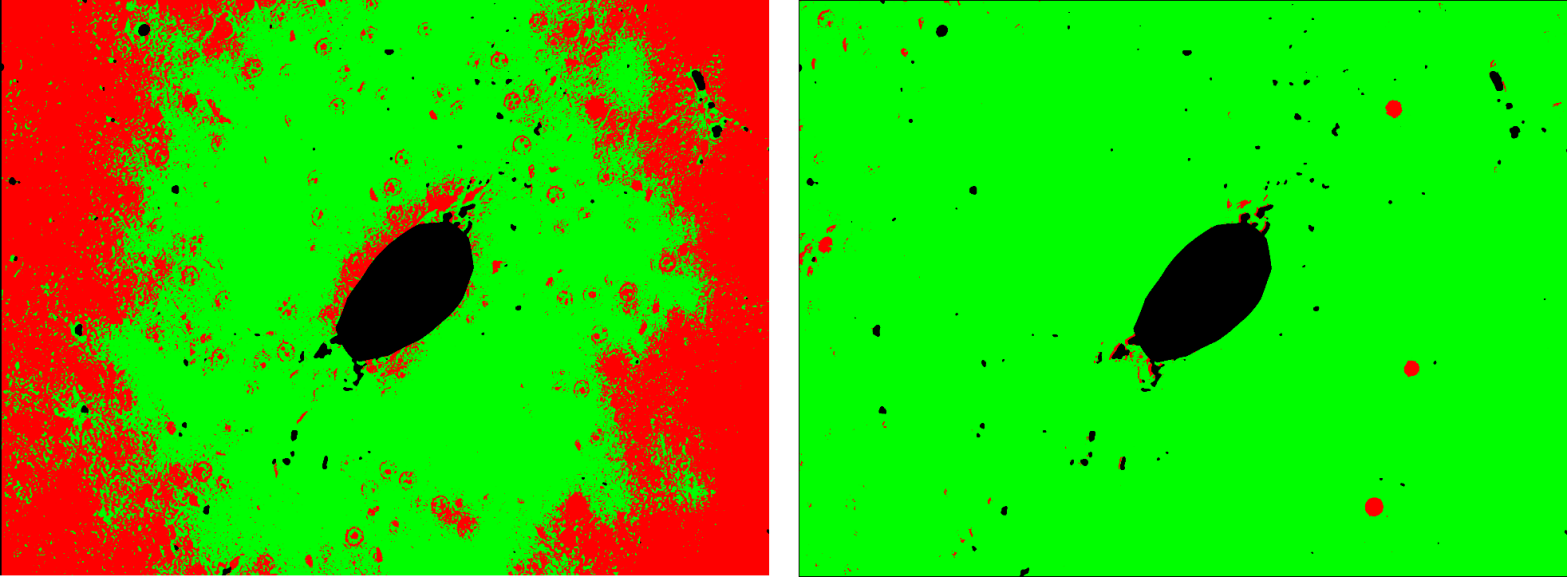
**Thresholding result without vein**. Results of Otsu thresholding (left) and EM (right) methods for the image in Figure 9 after vein exclusion. Otsu thresholding splits the background into two classes instead of separating cell and background classes, as the cell class is negligible. EM assigns all non-representative classes (cells and vein) to one Gaussian curve such that, after vein exclusion, the cell class is correctly extracted.

For a more adequate detection of the proliferating hepatocytes, we discard the vein region, which was detected in the red channel, by excluding the vein mask from the image histogram. In Figure 10, the results obtained without vein exclusion are presented. In Figure 11, the resulting threshold values obtained with Otsu thresholding and EM are presented overlaid with the respective histogram. As the cell class is negligible when compared to both vein and background classes or to the intensity variations inside the background class (see Figure 11), the Otsu thresholding method gives an incorrect result. In Figure 12, the results obtained with vein exclusion are shown. The result of EM method after vein exclusion, however, corresponds to the cell class in the initial image.

### Vein exclusion analysis

The preceding subsection shows that vein structures in the images make the classification task much tougher, but it also shows that vein exclusion can solve the problem. There is a number of works that tackle the problem of the vessel structure segmentation in CT scans [21,22]. However, the task of vein segmentation from histological images is hardly addressed in the literature. We included the venous structure segmentation as a part of our processing pipeline, utilizing automatic thresholding and morphological operations. Certainly, some more sophisticated techniques, such as, for example, active contours [6] can be applied, but we leave this point for future work.

**Figure 13:**
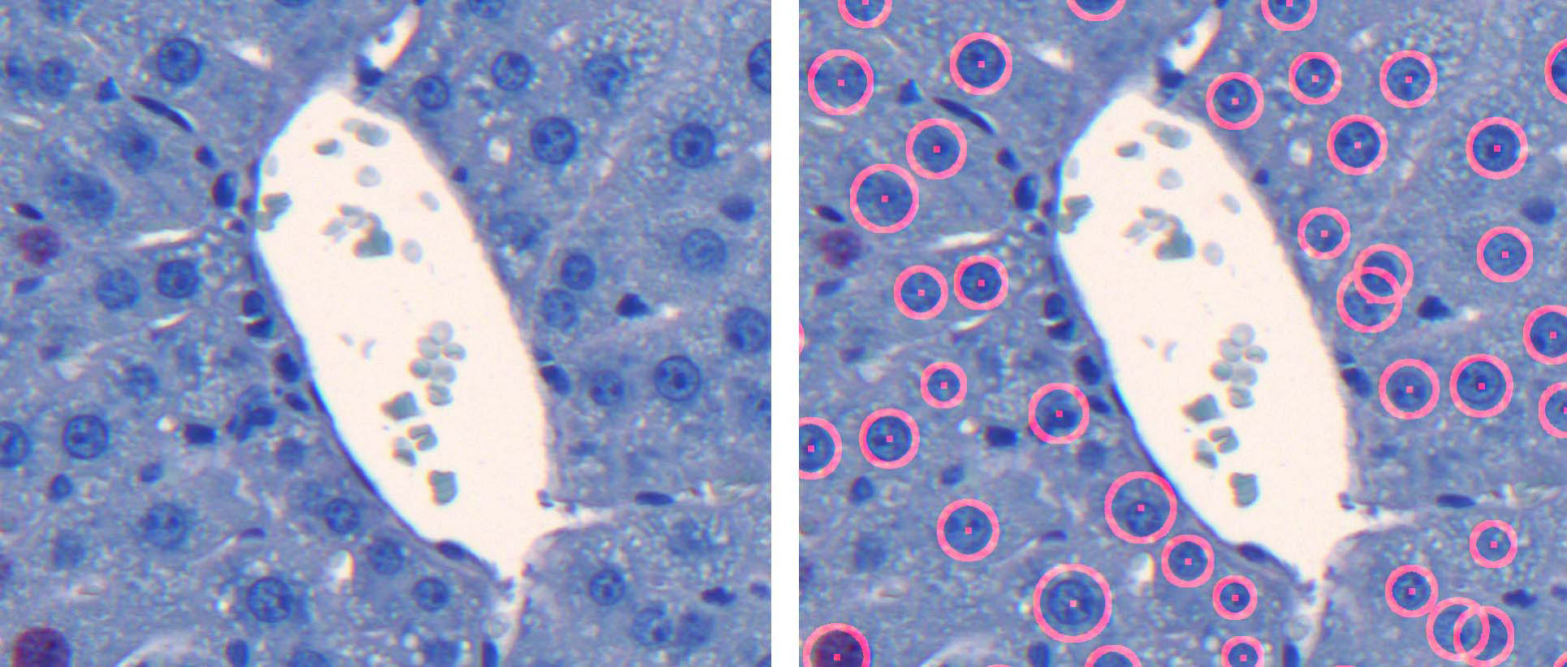
**Close-up view of vein: Simple case**. Close-up view of a vein region in image D5-CV1 (left). As the region is represented by a connected component, the Fill Holes filter suffices to generate the mask for vein exclusion. Detection result after vein exclusion (right).

If the vein region is represented by one connected component that encloses all the blood components, the Fill Holes filter removes the blood components and the resulting vein mask can be used to remove the vein structures from the image channels. A closeup view of such a vein region in image "D4-CV1" is shown in Figure 13.

**Figure 14:**
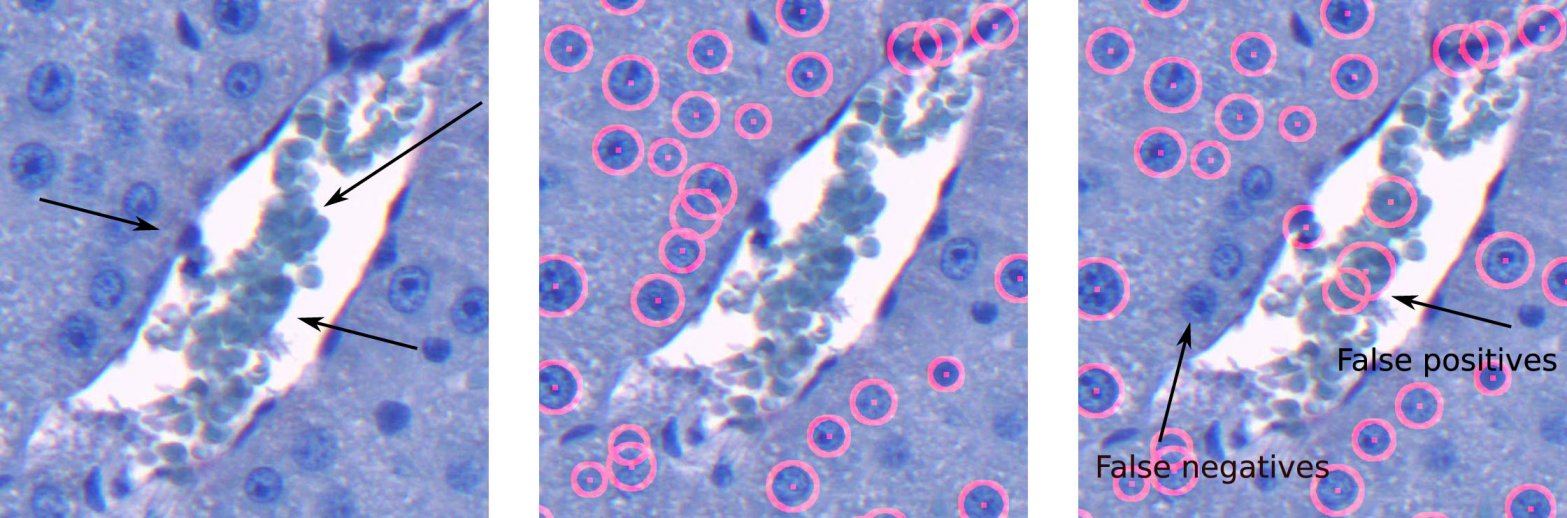
**Close-up view of vein: Complicated case**. Close-up view of the vein region of the image in Figure 2 (left), cell detection result after vein smoothing and elimination (middle), and cell detection result without prior vein handling (right). As the blood areas separate the vein into several components, the Fill Holes filter fails to remove all cellular components of the blood, see the areas marked with arrows (left). Vein smoothing and elimination allows for a correct detection (middle). If no vein exclusion is applied, the detection results in a number of additional false positives and false negatives (right).

If the vein region is represented by several components, the Fill Holes filter fails to remove all blood in the vein, which may lead to false positives. In Figure 14 (left), one can observe the close-up view of such a vein region from Figure 2. In such a case, we apply the vein smoothing step to build the vein mask. Figure 14 (middle) shows the correctly detected cells after vein smoothing and elimination in comparison to the result obtained without prior vein handling shown in Figure 14 (right).

In the current implementation, the presence of the vein structure and the correspondent number of classes in the thresholding step is indicated by the filename. In future work, we want to replace this convention with an automatic investigation of the presence of vein structures.

### General analysis

**Figure 15:**
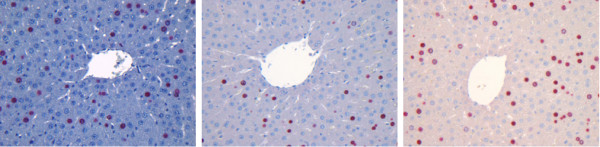
**Example images**. Images of stained sections are always subject to variations in the color properties especially when acquired from different experimental runs, samples, or animals. Images from three datasets D3-CV1, D7-CV1, and D8-CV1 are presented.

In Figure 15, images from three datasets with differences in color properties are shown. The results for the detection of all hepatocytes are presented in Figure 5 in an overlaid fashion. The circles around the cells represent the result obtained with our pipeline and correspond to the results shown in Table 2. Our approach is robust enough to deal with images with different color properties and gives results with high sensitivity (above 90%) and low FPF (below 15%).

As stated in Tables 2 and 3, the average sensitivity for the detection of all hepatocytes is 93% and for the detection of proliferating hepatocytes is 91%. The results that have been obtained with our pipeline lie within the expert dispersion ranges. All the results were obtained using the same set of parameters for all datasets. Although the datasets exhibit significant variations in color properties, no additional parameter adjustment was necessary.

Although the selected default algorithm settings work well for a wide range of images, we observed that some improvement of the results can still be obtained for certain images by fine-tuning the parameters. In general, to produce best results for a series of images with specific color properties, the user should process one "typical" ROI image from the sample, select the appropriate parameters for each step, and then apply the selected settings to the whole sample.

#### Complicated cases

**Figure 16:**
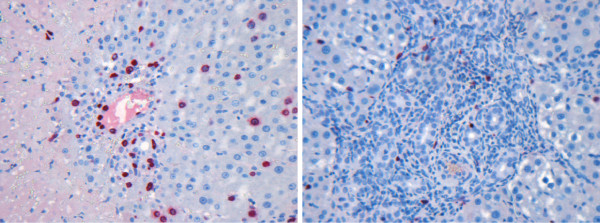
**ROIs with morphological complications**. ROI containing necrotic area (left) and ROI with severe bile duct proliferation (right).

Apart from testing our pipeline on two batches of ROIs with acceptable quality, that have been taken from the stained liver sections with normal morphology, we have also selected five more complicated cases. They can be separated into two categories. The first category contains the images with some morphological artifacts. In Figure 16, the images that belong to this category are shown.

**Figure 17:**
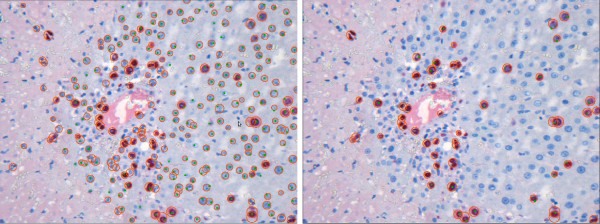
**Overlaid Results**. Overlaid detection results for the left image in Figure 16. The results are 88% sensitivity and 38% FPF for total HC (left) and 100% sensitivity and 56% FPF for proliferating HC (right). Green dots denote the expert groundtruth, circles are the result of our pipeline. Due to the necrotic area, the false positive rates are rather high.

There is a large necrosis area shown in Figure 16 (left image). The normal liver structure could not be preserved in the necrosis area. Moreover, there is a number of infiltrating cells around the preportal area, and some of them are similar to HC in size. This causes a certain number of false positives, as it is shown in Figure 17. The results for this image are 88% sensitivity and 38% FPF for the total HC, and 100% sensitivity and 56% FPF for the proliferating HC.

**Figure 18:**
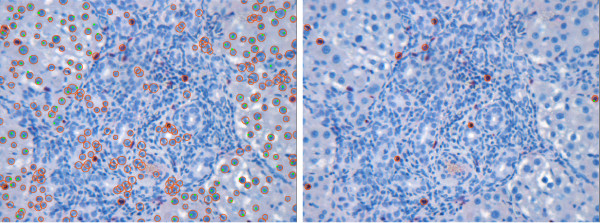
**Overlaid Results**. Overlaid detection results for the right image in Figure 16. It is problematic to differentiate the HC out from the bile epithelial cells even by manual counting. Green dots denote the expert groundtruth, circles are the result of our pipeline.

The liver structure shown in Figure 16 (right image) has been impaired by severe bile duct proliferation. It is hard to differentiate the HC out from the bile epithelial cells even by manual counting. The overlaid results are shown in Figure 18.

**Figure 19:**
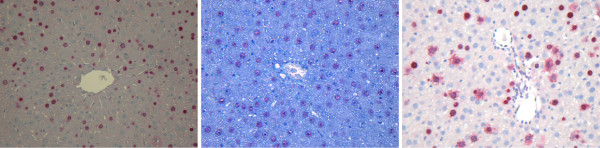
**ROIs with staining complications**. ROI with too reddish background and light blue HC (left); ROI with weakly stained proliferating HC, inhomogeneous and bright blue background and non-proliferating HC (center); ROI with bright background and blurry stained proliferating HC (right).

The images that have lower quality due to the staining settings belong to the second category (see Figure 19).

**Figure 20:**
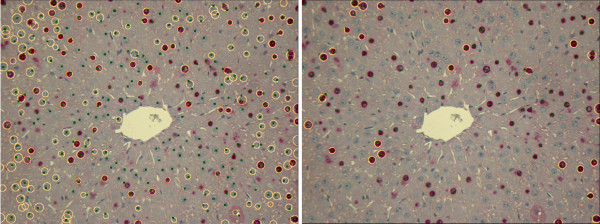
**Overlaid Results**. Overlaid detection results for left Figure 19. The results are 60% sensitivity and 10% FPF for total HC (left) and 32% sensitivity and 0% FPF for proliferating HC (right). Due to the weak staining of HC the false negative rates are high. Green dots denote the expert groundtruth, circles are the result of our pipeline.

In Figure 19 (left image), one can observe a ROI with a too reddish background and light blue HC. This causes many misses (false negatives) and results in low sensitivity. The overlaid results are shown in Figure 20. The results for this image are 60% sensitivity and 10% FPF for the total HC, and 32% sensitivity and 0% FPF for the proliferating HC.

**Figure 21:**
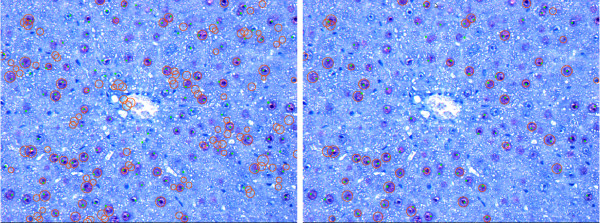
**Overlaid Results**. Overlaid detection results for central image Figure 19. The weak staining for positive cells and too bright and inhomogeneous background impairs the detection. The results are 36% sensitivity and 70% FPF for total HC (left) and 55% sensitivity and 22% FPF for proliferating HC (right). Green dots denote the expert groundtruth, circles are the result of our pipeline.

In Figure 19 (central image), the image with a rather weak staining for positive cells and too bright and inhomogeneous background. The non-proliferating hepatocytes are stained weakly and the boundaries of the cells are not pronounced. This results both in low sensitivity (36% for total HC and 55% for proliferating HC) and high false positive fraction (70% for total HC and 22% for proliferating HC). The overlaid results are shown in Figure 21.

**Figure 22:**
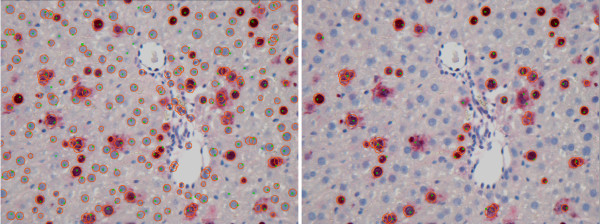
**Overlaid Results**. Overlaid detection results for the right image in Figure 19C. Due to the blurred proliferating HC and similarity in intensity of the background and venous structure the detection results have a rather high false positive fraction. The results are 90% sensitivity and 30% FPF for the total HC, and 80% sensitivity and 26% FPF for proliferating HC. Green dots denote the expert groundtruth, circles are the result of our pipeline.

In Figure 19 (right image), the image with a blurred staining of proliferating HC and bright background is shown. Due to the brightness of background the detection of the venous structure fails and a number of false positives is detected. The blurred staining of proliferating HC also causes high false positive detection rates. The overlaid results are shown in Figure 22. The detection results are 90% sensitivity and 30% FPF for the total HC, and 80% sensitivity and 26% FPF for proliferating HC.

Summarizing the observations shown above, we state, that our pipeline successfully determines the target cells when the contrast between them and the background is strong enough. However, the morphological abnormalities can severely impair the detection.

#### Comparison to other techniques

Visual analysis of cell samples has played a dominant role in the history of biology [23]. While numerous commercial and free software packages are available for image analysis, many of them are designed for a very specific purpose and data type (for example, [24]). Most commercial software is proprietary, which means that the underlying methods of analysis are hidden from the researcher.

We have chosen freely available, open-source image analysis software CellProfiler (http://www.cellprofiler.org, [23,25]) for comparison purposes. To detect the total number of HC we have applied a pipeline that is similar to the one proposed, for instance, in [25]. The pipeline consists of smoothing and object detection including size and roundness filters. The main part of that pipeline is IdentifyPrimAutomatic module. This module identifies primary objects (e. g., nuclei) in grayscale images that show bright objects on a dark background. It contains a three-step strategy based on a watershed algorithm [26] for separating the overlapping cells. In step one, CellProfiler determines whether an object is an individual nucleus or two or more clumped nuclei. In step two, the edges of nuclei are identified. For nuclei within the image that do not appear to touch, the edges are easily determined using thresholding. The clumped cells are divided using the distance-transformed watershed algorithm [26-28]. In step three, the objects smaller than a user-specified size range, are discarded. A more detailed description can be found on http://www.cellprofiler.org.

**Figure 23:**
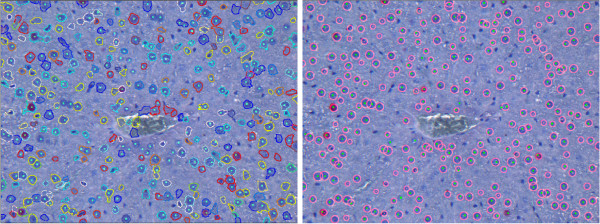
**Comparison**. Overlaid results of CellProfiler pipeline with watershed (left) and our pipeline (right) for image D2-CV1 are shown. The greenish dots denote the expert-defined groundtruth. Watershed (left image) successfully separates overlapping cells, but many of the false positives can be eliminated if more sophisticated size and roundness filters are applied. Our pipeline with Hough transformation and vein exclusion allows for the more exact target cells detection.

Such an approach is rather general and allows to quantify cells of any type and form. After the boundaries of the overlapping cells are found with the watershed method, the post-processing consisting of size and shape filters can be applied. We applied it to several images from our test datasets and obtained rather satisfactory results (for example, for image D2-CV1 sensitivity equals 90%, FPF equals 39%), but many of the false positives can be eliminated by some more sophisticated size and roundness filters. However, as our case is rather specific, i. e., the form of cells is known in advance, we assume, the application of the Hough transformation here allows for more exact quantification due to several reasons. First, in the process of evidence accumulation the regions that are not round enough are excluded by the relevance thresholding. Second, after the circles are found the information about radii and circle centers is used for additional analysis. This produces results with high sensitivity and low FPF, but at the same time increases the number of the pipeline parameters. In Figure 23, the overlaid results of CellProfiler pipeline with watershed (left) and our pipeline (right) for image D2-CV1 are shown. The greenish dots denote the expert-defined groundtruth.

## Conclusions

We have presented an algorithm for automatic hepatocyte detection, applied it to data of different characteristics, and compared the automatically calculated results to the manually detected ground truth. The proposed processing pipeline consists of a smoothing step, an automatic thresholding, a connected-component processing, vein exclusion (when necessary), and a Hough transformation. The default parameter values of the algorithm worked well for the data that we processed. In addition, the parameters can be adjusted in a semi-automatic manner and saved to a file such that they can be used in a fully automated batch processing.

The automatic processing of file series allows to produce the desired results in much shorter time when compared to the manual or semi-automatic single file processing. The proposed automatic pipeline gives results with high sensitivity and low false positive fraction for a wide range of images having different color properties. It can be used for the subsequent hepatocyte quantification not only in the selected ROIs, but also in the series of liver sections.

## Authors' contributions

TI developed the algorithm, implemented the software, run the main experiments, and drafted the manuscript. AS and AH participated in the algorithm development, and revised the manuscript. MD provided the datasets and the groundtruth evaluation. UD revised the manuscript, and provided the experimental samples. OD developed the 3D Histology strategy, and revised the manuscript. HH participated in the strategy development, and revised the manuscript. LL participated in the algorithm development, revised and rewrote the manuscript. All authors read and approved the final manuscript.
